# The Effect of IDO on Neural Progenitor Cell Survival Under Oxygen Glucose Deprivation

**DOI:** 10.3389/fncel.2020.581861

**Published:** 2020-10-30

**Authors:** Jixian Wang, Brian Wang, Lei Jiang, Kaijing Zhou, Guo-Yuan Yang, Kunlin Jin

**Affiliations:** ^1^Department of Rehabilitation, Ruijin Hospital, Shanghai Jiao Tong University School of Medicine, Shanghai, China; ^2^Department of Pharmacology and Neuroscience, University of North Texas Health Science Center Fort Worth, Fort Worth, TX, United States; ^3^Med-X Research Institute and School of Biomedical Engineering, Shanghai Jiao Tong University School of Medicine, Shanghai, China; ^4^Department of Neurology, Ruijin Hospital, Shanghai Jiao Tong University School of Medicine, Shanghai, China

**Keywords:** IDO, neural progenitor cells, oxygen glucose deprivation, cell viability, leptin

## Abstract

**Objective**: Indoleamine 2,3-dioxygenase (IDO) activity plays an important role in many neurological disorders in the central nervous system, which may be associated with immunomodulation or anti-inflammatory activity. However, the action of IDO in the ischemic condition is still poorly understood. The purpose of the present study is to explore the expression and action of IDO in stem cell culture under oxygen and glucose deprivation.

**Methods**: Neural progenitor cells were obtained from the human embryonic stem cell line BG01. These cells underwent oxygen and glucose deprivation. We examined the IDO expression at 3 and 8 h of oxygen and glucose deprivation and then examined neuronal progenitor cell viability in the normal and oxygen and glucose deprivation condition using the [3-(4,5-dimethylthiazol-2-yl)-2,5-diphenyltetrazolium bromide] assay. In addition, we studied the effect of IDO inhibition and the expression of TNF-α, IGF-1, VEGF, IL-6, FGFβ, TGFβ, EGF, and Leptin to explore the mechanism of IDO under the oxygen and glucose deprivation.

**Results**: IDO expression in neural progenitor cells increased under oxygen and glucose deprivation, which is closely associated with cell death (*p* < 0.05). Inhibiting IDO did not affect cell survival in normal neural progenitor cells. However, inhibiting IDO could attenuate cell viability under oxygen and glucose deprivation (*p* < 0.05). Further study demonstrated that IDO expression was closely associated to the growth factor’s leptin expression.

**Conclusions**: Our results demonstrated that an increase of IDO under oxygen and glucose deprivation was associated with cell death, suggesting that inhibiting IDO could be a target for neuroprotection.

## Introduction

Ischemic stroke is one of the leading causes of death and adult disability worldwide, which leads to massive cell death and complex pathological changes (Tang et al., 2016; Naghavi et al., 2017; Wang et al., 2017). With the recent improvement of acute ischemic stroke management, tPA induced thrombolysis and intravascular thrombectomy are likely to provide insights to develop therapeutic strategies that may benefit stroke patients (Yang et al., 2019). However, stroke morbidity and mortality are still high. To understand the mechanisms of stroke occurrence and development is extremely critical to reduce the imminent danger of stroke. Increased evidence indicated immunomodulation and inflammation is crucial in the physiopathological development of stroke (Jayaraj et al., 2019). Our previous studies demonstrated that during an ischemic stroke attack, resident microglia/macrophages were activated, which could increase cytokines such as IL-1β, IL-6, TNFα, chemokines CXC, CC, CX3C, and XC, and adhesion molecules, calcium-independent integrins, and calcium-dependent cadherins expression in the ischemic region as well as system blood circulation (Ma et al., 2017). In addition, activated macrophages/microglia also triggered the immune system cascade through activating regulatory T cells, T cells, B cells, and the complement system (Wang et al., 2015). However, we do not know how these inflammatory or immune responses reflect the progression of these events during ischemic brain injury. A recent study in a mouse model of middle cerebral artery occlusion demonstrated that the macrophages/microglia could enhance indoleamine 2,3-dioxygenase 1 (IDO1)-dependent neurotoxic kynurenine metabolism during ischemic pathogenesis, which was closely related to the post-stroke depression (Koo et al., 2018). However, the role of IDO in the cell death under oxygen and glucose deprivation is largely unknown.

The kynurenine pathway is initiated by the oxidative metabolism of tryptophan. It has been studied since the early 20th century. No specific neurobiological activity was founded for kynurenine and its catabolic products. In 1978, a study showed that several tryptophan metabolites could produce convulsions when injected directly into the brain (Lapin, 1978). In the 1980s, there was interest in the biological function of an initial enzyme IDO in the kynurenine pathway, which could convert tryptophan to kynurenine. Pfefferkorn (1984) observed that IDO was activated by interferon in the immune response to infection, inhibiting the harmful effects of infectious mediators. However, the mechanism of the anti-infective effect was caused by the depletion of tryptophan or by the accumulation of kynurenine and its downstream metabolites was still controversial (Badawy, 2017). IDO is a heme-containing enzyme expressed in a number of tissues and cells (Yamazaki et al., 1985). IDO is an important molecule of the immune system and plays a part in the natural defense against various pathogens (Yoshida and Hayaishi, 1978; Yoshida et al., 1979). IDO has a function in the response to the inflammatory response and has an immunosuppressive function to limit T cell function (Munn and Mellor, 2013). IDO not only plays a key role in suppressing the anti-tumor immune response in the body (Prendergast et al., 2014), but is also involved in many neurological diseases such as atherosclerosis (Cole et al., 2015), Huntington’s disease (Perkins and Stone, 1983), Alzheimer’s disease, multiple sclerosis (Fiala et al., 1998; Guillemin et al., 2003), and psychiatric disorders (Savitz et al., 2015; Erhardt et al., 2017). However, the effect of IDO on these neurological disorders is still debatable or just beginning to receive a small degree of attention (Schwarcz and Stone, 2017). The function of IDO during cerebral ischemia has not been well studied both *in vivo* and *in vitro*. In the present study, we examined the IDO expression in neural progenitor cells during oxygen and glucose deprivation to explore the function of IDO in the neuronal death process. We further studied the relationship between IDO expression and the inflammatory response.

## Materials and Methods

Research protocol was approved by the Institutional Animal Care and Use Committee (IACUC) of Shanghai Jiao Tong University, Shanghai, China. The experiments were performed under the ARRIVE guideline.

### Cell Culture and Identification

Neural stem/progenitor cells (NPCs) derived from the human embryonic stem cells (*h*ESC) line BG01 were obtained from Aruna Biomedical (Athens, GA, USA). Cells were seeded on 0.01% polyornithine (Sigma–Aldrich, St. Louis, MO, USA) and 20 μg/ml laminin (Stemgent Inc., Cambridge, MA, USA) coated dishes and cultured in a medium, consisting of a neurobasal medium with a B27 supplement containing 2 mmol/l L-glutamine (both from Gibco Life Sciences, Grand Island, USA), 50 μg/ml Pen/Strep (Invitrogen, Carlsbad, CA, USA), 10 ng/ml leukemia inhibitory factor (Thermo Fisher Scientific, Waltham, MA, USA), and 20 ng/ml fibroblast growth factor-2 (R&D Systems, Minneapolis, MN, USA; Jin et al., 2010). Cells were cultured in a humidified incubator at 37°C with 5% CO_2_. They were propagated in the medium and, on reaching 90% to 100% confluence, were triturated to detach them from the dish.

### Immunofluorescent Staining

Cells were treated with 100% methanol (chilled at −20°C) for 5 min. Then cells were washed with ice-cold PBS. The cells were incubated with 1% BSA in PBS for 60 min to block any unspecific binding of the antibodies. Then the cells were incubated in the primary antibodies Nestin (1:200 dilution, Millipore) and SOX2 (1:100 dilution, GeneTex, Zeeland, MI, USA), respectively at 4°C overnight, followed by incubation with secondary antibodies (Life Technologies) for 1 h at room temperature. The results were observed under confocal microscopy (Zeiss, Thornwood, NY, USA). The photomicrographs were taken for cell identification.

### Oxygen-Glucose Deprivation (OGD)

The procedure of oxygen-glucose deprivation (OGD) and reoxygenation was as follows: NPCs were seeded on 0.01% polyornithine (Sigma–Aldrich) and 20 μg/ml laminin (Stemgent) coated dishes and cultured. Cells were transferred from the original media to the deoxygenated glucose-free neurobasal medium. The OGD and reoxygenation experiment was performed using a specialized sealed chamber, which contained an anaerobic gas mixture (95% N_2_ and 5% CO_2_) at 37°C. NPCs cultured in the NPC culture medium without OGD were used as a negative control. After 3 or 8 h treatment, these cells were removed from the chamber; and the medium in the cultures was replaced with the maintenance medium. In the 8-h OGD groups, cells were then allowed to recover for 24 h in a regular incubator.

### Western Blot Analysis

Cells were collected and lysed in RIPA (Millipore, Burlington, USA) supplemented with a cocktail (Sigma–Aldrich, St. Louis, MO, USA), 1 mmol/l PMSF (Thermo), and a phosphatase inhibitor (Thermo). For Western blots analysis, denatured samples containing the same amount of proteins (30 μg) were loaded onto the resolving gel (EpiZyme, Shanghai, China) for electrophoresis. Proteins were then transferred onto a nitrocellulose membrane (Whatman, Piscataway, NJ, USA). The membrane was blocked with blocking buffer (EpiZyme) and then incubated with primary antibodies at the following dilution IDO (1:200 dilution, LifeSpan Biosciences, Seattle, WA, USA) at 4°C overnight, respectively. The membrane was washed, incubated with the appropriate HRP-conjugated secondary antibody for 1 h, and then reacted with enhanced chemiluminescence substrate (Pierce, Rockford, IL, USA). The results were recorded by the Quantity One image software (Bio-Rad, Hercules, CA, USA) and relative intensity was calculated using the ImageJ software (NIH, USA).

### MTT Assay

Cell survival assays were performed using an MTT kit (Sigma–Aldrich, St. Louis, MO, USA). The MTT solution was added to each well and incubated at 37°C for 4 h. After incubation, MTT solvent was added into each well. The plate was wrapped in foil and shaken on an orbital shaker for 15 min. Occasionally, pipetting of the liquid was required to fully dissolve the MTT formazan. Absorbance was measured at 590 nm using a microplate reader (Thermo Fisher Scientific, Waltham, MA, USA).

### ELISA Assay

To assess the release of angiogenesis-related cytokines in *h*ESC derived NPCs after OGD, and 1-MT treatment, the amount of released TNF-α, IGF-1, VEGF, IL-6, FGF-β, TGF-β, EGF, and Leptin was measured by using a human angiogenesis ELISA kit (Signosis Inc., Santa Clara, CA, USA). The supernatant was collected from each sample. A hundred microliter sample was added into the well and incubated, for 1 h at room temperature with gentle shaking. Each well was washed by adding 200 μl of 1× assay wash buffer three times. A total of 100 μl of diluted biotin-labeled antibody mixture and 100 μl of diluted streptavidin-HRP conjugate were in turn added to each well and incubated respectively for 1.5 h at room temperature with gentle shaking. A total of 100 μl of substrate was added to each well and incubated for 30 min. A total of 50 μl of stop solution was added to each well. The optical density was determined by a microplate reader at 450 nm within 30 min.

### Statistical Analysis

Quantitative data were presented as mean ± SD and compared by one-way and two-way analysis of variance (ANOVA) with repeated measures, followed by *post hoc* multiple comparison tests (Fisher PLSD or Student’s paired *t*-test with the Bonferroni correction) using the SPSS software (v18.0, SPSS Inc., Chicago, IL, USA). A probability value of less than 0.05 was considered to represent statistical significance.

## Results

### NPC Identification and Culture

**Figure 1:**
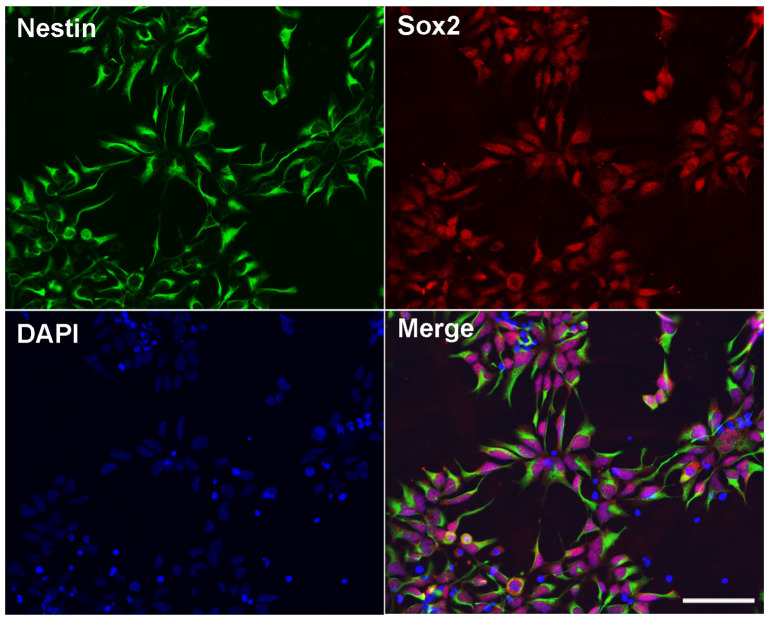
Identification of human embryonic stem cells (*h*ESC)-derived neural progenitor cells (NPCs). Immunostaining images of *h*ESC-derived NPCs showed anti-Nestin (green), Sox2 (red), and DAPI (blue). Scale bar = 50 μm.

*h*ESC line BG01-derived NPCs were cultured and maintained in a medium. The morphology of the NPCs were characterized by a double immunostaining with anti-Nestin and anti-Sox2. We found that more than 99% of cells expressed both Nestin and Sox2 (Figure 1), which indicated that these cells showed the NPC morphology and could be appropriately used in the subsequent experiments.

### IDO Expression Increased in the NPCs After OGD

**Figure 2:**
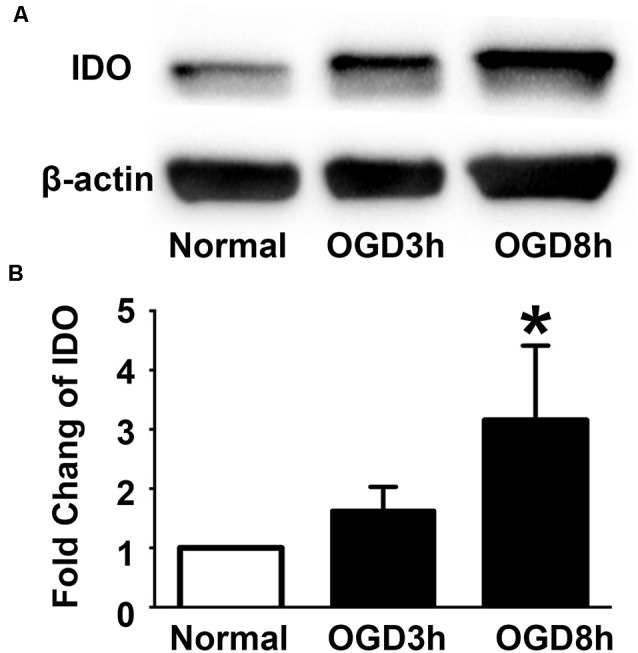
Indoleamine 2,3-dioxygenase (IDO) expression increased in the NPCs after oxygen glucose deprivation (OGD). **(A)** Western blot results showed that the expression of IDO after 3 and 8 h of OGD in *h*ESC-derived NPCs. β-actin was an internal control. **(B)** The bar graph represented the semi-quantification of IDO expression. *N* = 5 per group, *p < 0.05, compared to the control.

To examine the expression of IDO in the NPCs during oxygen and glucose deprivation, we performed an NPCs OGD model. Western blot results showed that IDO expression was significantly increased in NPCs after 3 and 8 h of OGD compared to the NPCs that were in a normal condition (Figure 2, *p* < 0.05). There was the potential that IDO expression would increase with time.

### Inhibition of IDO Attenuated NPC Viability

**Figure 3:**
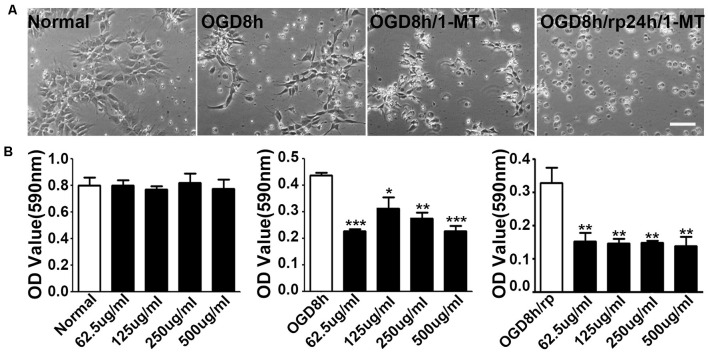
Inhibition of IDO attenuated NPC viability. **(A)** Photomicrographs taken with a phase contract microscope showed neuroepithelial (NEP) morphological changes after 8-h OGD, 8-h OGD plus 1-MT treatment, and 8-h OGD followed by 24-h reperfusion plus 1-MT treatment. Scale bar = 50 μm. **(B)** Bar graphs presented the MTT results: the effect of 1-DL-MT at 62.5, 125, 250, and 500 μg/ml on NEP cells under normal condition (left), after 8-h OGD (middle), and 8-h OGD plus 24-h reperfusion (right). *N* = 5 per group, *p < 0.05, **p < 0.01, ***p < 0.01, compared to the controls.

1-MT is an inhibitor of IDO. To explore the effect of IDO on the viability of NPCs, 1-MT was used to inhibit IDO expression. As is shown in the Figure 3A, some of the NPCs began to die after 8 h of OGD. 1-MT treatment further exacerbated the damage of the NPCs. Using the MTT method, we found that 1-MT had no effect on the survival in normal NPCs (Figure 3B, left, *p* > 0.05). However, 1-MT significantly reduced the viability of NPCs after 8 h of OGD at 62.5–500 μg/ml concentrations (Figure 3B, middle, *p* < 0.05 to *p* < 0.001). Moreover, 1-MT had the same effect on the viability of NPCs after 8 h of OGD and 24 h reperfusion (Figure 3B, left, *p* < 0.01).

### IDO Inhibition Reduced Cytokine Expression

**Figure 4:**
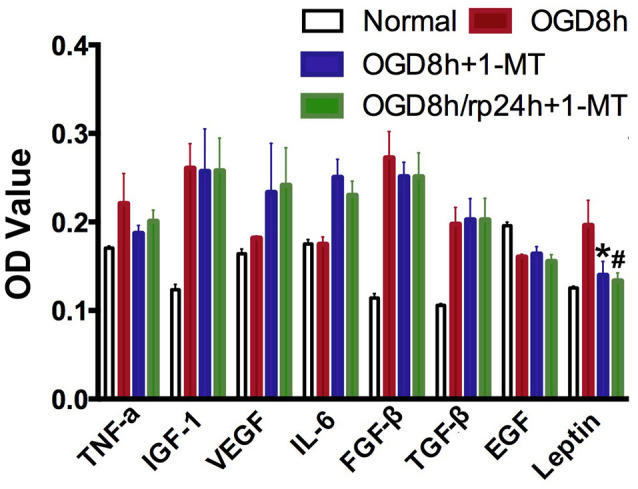
IDO inhibition reduced cytokine expression. Bar graphs presented that TNF-α, IGF-1, VEGF, IL-6, FGFβ, TGFβ, EGF, and Leptin expression in the OGD by ELISA assay. *N* = 5 per group, *p < 0.05, OGD 8 h vs. OGD 8 h + 1-MT group; ^#^*p* < 0.05, OGD 8 h vs. OGD 8 h/rp 24 h + 1-MT.

To further explore the molecular mechanism of IDO, ELISA was applied to detect cytokine expression in OGD-treated NPCs. As shown in Figure 4, we found that after 8 h of OGD, IGF-1, FGF-β, TGF-β, and Leptin expression in NPCs were significantly increased compared to the normal NPCs (*p* < 0.01). However, only Leptin was significantly decreased after 8 h of OGD with 1-MT treatment (*p* < 0.05). This phenomenon was also observed in the group of 8 h of OGD and 24 h reperfusion with 1-MT treatment (*p* < 0.05).

## Discussion

Although IDO plays an important role in many neurological diseases such as Huntington’s disease, Alzheimer’s disease, multiple sclerosis, and psychiatric disorders, the effect of IDO in the ischemic neuronal injury is largely unknown. In the present study, we used an OGD-treated NPC-cultured model *in vitro* which demonstrated that: (1) the expression of IDO in NPCs increased under the OGD condition, which was closely related to cell survival; (2) the inhibition of IDO could attenuate NPC viability; and (3) increased IDO is closely associated to growth factors TNFα, FGFβ, and Leptin expression. Our results suggested that inhibiting IDO could be a potential approach for neuroprotection in future studies *in vivo*.

After the discovery of IDO, the kynurenine pathway and its roles in the nervous and immune systems development was evaluated. IDO was involved not only in primarily peripheral afflictions including arthritis and atherosclerosis but also in central inflammation and immune function in Alzheimer’s disease, multiple sclerosis, and Huntington’s disease. These interactions could contribute to a physiologic and pathogenic role to the immune system activity with potentially common targets. IDO could be activated in astrocytes, microglia, and even in the neurons (Guillemin et al., 2003, 2005). However, if IDO was expressed in NPCs it was not well understood. An interesting topic of increasing attention is the link between kynurenines and stem cell biology. Human and mouse mesenchymal stem cells (MSCs) and neural stem cells (NSCs) expressed the kynurenine pathway (Croitoru-Lamoury et al., 2011). Pluripotent progenitor cells had IDO-dependent immunomodulatory properties, implicating kynurenine, and possibly its downstream metabolites (Jacobs et al., 2013). Numerous studies demonstrated that MSC transplantation had neuroprotection and neuronal repair function, which was a new clinical approach for repairing injured tissue. The function of MSCs could modulate immunological responses *via* T cell suppression, through IDO and toll-like receptor (TLR) signaling pathways (Lyakisheva et al., 2002; Aggarwal and Pittenger, 2005). In this study, we chose NPCs to explore the effect of IDO in brain neurons because NPCs were an important component during injured brain tissue repair. We found that, similar to the MSCs, IDO expression increased in NPCs during OGD, which is related to the cell viability. Increased IDO could attenuate NPC death during OGD, suggesting that IDO plays a neuroprotection role in the cell survival process.

In studies of the role of kynurenines in the brain, the most useful information was commonly collected from *in vivo* models or acutely prepared tissue slices, while freshly dissociated cells *in vitro*, for example, neurons, astrocytes and microglia also provided relevant information (Guillemin et al., 2007). In a mouse middle cerebral artery occlusion model, Koo et al. (2018) found that microglial IDO expression, QUIN production, and reactive oxidative species (ROS) were prominently increased in the accumbens nucleus, hippocampus, and hypothalamus, and found that these increases were related to mouse depressive-like behaviors. They demonstrated that adjunctive antidepressant aripiprazole ameliorated depressive behavior and cognitive impairment in the ischemic mice *via* downregulation of IDO1, HAAO, QUIN, and ROS. The action of IDO could be sustained for at least 9 weeks. This *in vivo* study provided evidence that the IDO1-dependent neurotoxic kynurenine metabolism represented a potential therapeutic target for the treatment of post-stroke dementia. However, our result was different from their study. We found that the over-expression of IDO could enhance cell viability while inhibiting IDO action could attenuate NPC survival. We believe that the effect of IDO in the early phase or the later phase of ischemia could be different. In the early stage, IDO may be involved in the acute inflammatory response while in the later stage it may promote anti-inflammation and immune-regulation. The action of IDO in the long term of neurological diseases could also be different with acute cerebral vascular disease. In addition, an argument against a special role of NMDA receptor inhibition could not be duplicated for the action of kynurenic acid in several experiments *in vitro* and *in vivo* (Hilmas et al., 2001; Beggiato et al., 2013), suggesting that IDO was involved in several signal pathways. Nevertheless, the effect of IDO on ischemic neurons and the brain should to be further studied.

Growing evidence indicated that IDO, TGF-β1, and PGE2 may represent relevant mediators of NK cell functional inhibition (Di Nicola et al., 2002; Meisel et al., 2004; Aggarwal and Pittenger, 2005). Quantitative real-time PCR confirmed that TNF-α could significantly upregulate HGF mRNA (English et al., 2007). Another study neutralized HGF and TGF-β1 and found that HGF worked in synergy with TGF-β1 to resist T cell recognition (Di Nicola et al., 2002). All this suggested that IDO and TGF-β1 together regulate the immune effect of NPCs. We found that increased IDO is closely associated to the cytokine TNF-α, and growth factors FGF-β and Leptin, but not IGF-1, VEGF, IL-6, TGF-β, and EGF expression. This finding indicated that IDO is not an exclusive mechanism for NPC protection especially in the early stage of ischemia.

In conclusion, our study demonstrated that IDO expression increased in NPCs during OGD, which is associated with NPC viability. IDO may represent a potential valuable target for the treatment of injured NPCs.

## Data Availability Statement

The raw data supporting the conclusions of this article will be made available by the authors, without undue reservation.

## Ethics Statement

The animal study was reviewed and approved by the Institutional Animal Care and Use Committee (IACUC) of Shanghai Jiao Tong University.

## Author Contributions

JW and KJ designed the experiment. JW performed all experiments, analyzed the data, and drafted the manuscript. BW, LJ, and KZ performed the Western blot and ELISA experiment. G-YY was responsible for supervising all the experimental designs, experimental performance, and finalizing the manuscript. All authors critically reviewed the manuscript. All authors contributed to the article and approved the submitted version.

## Conflict of Interest

The authors declare that the research was conducted in the absence of any commercial or financial relationships that could be construed as a potential conflict of interest.

The handling editor declared a past co-authorship with one of the authors KJ.

## Abbreviations

EGF: epidermal growth factor
1-MT: 1-Methyltryptophan
FGF-β: fibroblast growth factor
IDO: indoleamine 2,3-dioxygenase
IGF-1: insulin-like growth factor 1
IL-6: interleukin-6
MTT: [3-(4,5-dimethylthiazol-2-yl)-2,5-diphenyltetrazolium bromide
NPCs: neural progenitor cells
OGD: oxygen glucose deprivation
TGF-β: transforming growth factor
TNF-α: tumor necrosis factor α
VEGF: vascular endothelial growth factor.
